# Structural Framework for Analysis of CD4+ T-Cell Epitope Dominance in Viral Fusion Proteins

**DOI:** 10.1021/acs.biochem.3c00335

**Published:** 2023-08-09

**Authors:** Samuel J. Landry, Ramgopal R. Mettu, Jay K. Kolls, Judith H. Aberle, Elizabeth Norton, Kevin Zwezdaryk, James Robinson

**Affiliations:** † Department of Biochemistry and Molecular Biology, Tulane University School of Medicine, New Orleans, Louisiana 70112, United States; ‡ Department of Computer Science, Tulane University, New Orleans, Louisiana 70118, United States; § John W. Deming Department of Internal Medicine, Center for Translational Research in Infection and Inflammation, Tulane University School of Medicine, New Orleans, Louisiana 70112, United States; ∥ Center for Virology, Medical University of Vienna, 1090 Vienna, Austria; ⊥ Department of Microbiology & Immunology, Tulane University School of Medicine, New Orleans, Louisiana 70112, United States; # Department of Pediatrics, Tulane University School of Medicine, New Orleans, Louisiana 70112, United States

## Abstract

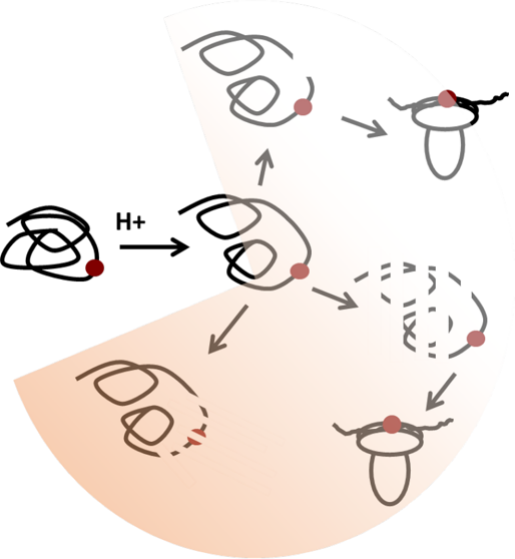

Antigen conformation shapes CD4+ T-cell specificity through mechanisms of antigen processing, and the consequences for immunity may rival those from conformational effects on antibody specificity. CD4+ T cells initiate and control immunity to pathogens and cancer and are at least partly responsible for immunopathology associated with infection, autoimmunity, and allergy. The primary trigger for CD4+ T-cell maturation is the presentation of an epitope peptide in the MHC class II antigen-presenting protein (MHCII), most commonly on an activated dendritic cell, and then the T-cell responses are recalled by subsequent presentations of the epitope peptide by the same or other antigen-presenting cells. Peptide presentation depends on the proteolytic fragmentation of the antigen in an endosomal/lysosomal compartment and concomitant loading of the fragments into the MHCII, a multistep mechanism called antigen processing and presentation. Although the role of peptide affinity for MHCII has been well studied, the role of proteolytic fragmentation has received less attention. In this Perspective, we will briefly summarize evidence that antigen resistance to unfolding and proteolytic fragmentation shapes the specificity of the CD4+ T-cell response to selected viral envelope proteins, identify several remarkable examples in which the immunodominant CD4+ epitopes most likely depend on the interaction of processing machinery with antigen conformation, and outline how knowledge of antigen conformation can inform future efforts to design vaccines.

## Introduction

Rarely has immunity to infectious disease so clearly depended on features of antigen processing, as is now becoming clear for COVID-19. Early studies suggested that the pathological mechanisms involved inappropriate or poorly timed CD4+ T-cell responses. CD4+ T cells protect the host by multiple mechanisms. They secrete molecules that stimulate growth, differentiation, and trafficking of other cells, including B cells and CD8+ T cells.^1,2^ CD4+ T cells also induce innate immune resistance in many other cell types that respond to interferon γ.^3^ Lastly, CD4+ T cells can directly kill pathogens and infected host cells by secreting cytolytic proteins and displaying death-inducing signaling proteins.^4^

New evidence indicates that infection early in the pandemic sets up CD4+ T-cell immune imprinting that reduces vaccine breadth against divergent variants of concern (VOC), such as omicron.^5^ A basic science rationale for these observations is beginning to emerge. The finding that responses to intact protein report differences in immune history led investigators to highlight antigen processing. This analysis outlines the degree to which conformational stability modulates antigen processing and raises the possibility that innate immunity shapes CD4+ T-cell specificity and response through the interaction of the antigen conformation and antigen processing.

## Mechanisms of CD4+ Epitope Dominance

### MHCII Polymorphism

Basic mechanisms of antigen presentation by major histocompatibility (MHC) class I and II pathways have been established for decades. Nevertheless, the success of predicting epitopes restricted by MHC class I has not been realized for predicting epitopes restricted by MHC class II. Differences in the pathways at multiple levels increase the diversity of the MHCII–peptide complexes. For the MHC class I (MHCI) pathway, well-defined peptides of eight to nine residues emerge from the cytoplasmic proteasome, pass through a transport apparatus into the endoplasmic reticulum, and fold with the MHCI molecule, which is then delivered to the cell surface. For the MHC class II (MHCII) pathway, broad clusters of peptides emerge from a seemingly chaotic mixture of partially folded antigens, proteases, and fully assembled MHCII molecules. For MHCI, the peptide-binding groove has deep pockets for the N- and C-termini and certain side chains. For MHCII, the ends of the groove are open; pockets are shallow, and hydrogen bonds to the peptide backbone provide the main stabilizing contacts.^6^ Whereas the identity of an MHCI ligand is usually unambiguous, the MHCII ligand is a cluster of overlapping sequences that sometimes bind in multiple registers, such that distinct surfaces are displayed to the T cells.^7,8^

T-Cell epitope maps for humans and other outbred animals have been difficult to interpret due to the extreme polymorphism of MHC proteins and the resulting diversity of peptide binding specificity. Scores of different class I and II alleles are represented in most populations. Given that there are six MHCI loci and three MHCII loci and chromosomes from two parents, individuals may express many different MHCI and MHCII molecules. With knowledge of the MHCI alleles present from DNA sequencing, one can predict an antigen’s MHCI-restricted epitopes with good accuracy, but the prediction of an antigen’s MHCII-restricted epitopes is more complicated.

Studies of the peptide specificity of particular MHCII molecules and the epitope prediction tools based on them have been developed.^9−17^ Some recent prediction efforts have sought to evaluate sequences outside of the MHCII-binding region that are thought to influence proteolytic processing.^18,19^ Others have considered the impact of self-antigen presentation on naïve T-cell frequencies.^20^ In spite of a very broad range of binding specificity and a relatively low threshold for adequate affinity, dominant CD4+ epitopes have been found to cluster in narrow regions of antigen sequences.^21,22^ The following aspects of the MHCII pathway presentation most likely constrain the breadth of specificity.

### DM-Catalyzed Peptide Exchange

The peptide-exchange catalyst DM (also known as HLA-DM in humans) accelerates peptide exchange through its interactions with susceptible MHCII molecules. Contacts near one end of the MHCII peptide-binding groove promote opening of the groove, resulting in faster on and off rates and therefore more opportunities for binding by higher-affinity peptides.^23−25^ The first, most important exchange reaction replaces the CLIP peptide fragment of the invariant chain, which associates with the newly synthesized MHCII until it is digested in the antigen-processing compartment.^26^ DM-catalyzed peptide exchange is itself regulated by the dedicated inhibitor molecule DO.^27^ At low pH, DO becomes unstable, relieving the inhibition of DM.^27^ The action of DM results in the focusing of T-cell responses onto fewer epitopes, which have higher affinity.^28,29^ We note that DM modulates presentation through its effects on the population of MHCII-bound species, which is affected by the concentration-dependent on rate, as well as the affinity-dependent off rate.^30,31^ Thus, the amount of a given epitope peptide emerging from antigen processing could be an important factor in DM-regulated epitope dominance.

A complex aspect of the DO–DM-regulated peptide exchange is created by the dynamic compartmentalization of processed antigen, MHCII, and DM. One study evaluated antigen presentation as a function of the ratio of free (uninhibited) DM to MHCII.^32^ In the context of B cells, a wave of antigen processing and presentation was initiated by internalization of antigen BCR and coordinated with the downregulation of inhibition by DO.

### Antigen Processing

Protein antigens must undergo proteolytic processing to make the peptide segments available for binding to MHCII proteins. For class II pathway processing, proteolysis occurs in an endosome–lysosome compartment simultaneously with MHCII maturation and peptide loading. Antigen segments become exposed by partial unfolding in the acidified endolysosomal compartment and by proteolytic nicking at conformationally flexible sites. Gradual exposure of antigen segments through unfolding and fragmentation, combined with recurrent sampling by DM-catalyzed peptide exchange in the MHCII-binding site, ensures that epitopes are presented in a hierarchical pattern that depends on both the antigen three-dimensional structure and peptide–MHCII affinity.

The folded conformation of the antigen is a barrier to proteolytic cleavage and peptide loading because an antigen segment must be in an extended conformation to form backbone hydrogen bonding with the protease active site or with the MHCII peptide-binding groove. Biochemists have long recognized the ability of protein structure to limit access to proteases and the fact that preferred sites of proteolytic attack occur in segments characterized by conformational dynamics. Despite having sequence preferences at the site of cleavage, many proteases cleave in the same region of a given folded protein, and denaturation of the protein removes this clustering of cleavage sites. In the original example of citrate synthase, the same disordered region is cleaved by four different proteases, and the clustering was explained by the tendency of proteases to cleave in the “hinges” and “fringes”.^33^ Studies of the conformational dynamics by nuclear magnetic resonance (NMR) and amide-group hydrogen–deuterium exchange have revealed that disordered protein segments exhibit motions on an extremely wide range of amplitudes and time scales, spanning from crankshaft rotations in nanoseconds to domain-wise unfolding in tens of seconds.^34,35^ The conformational dynamics themselves have been simulated computationally using all-atom models for short time scales and thermodynamic models of segmental protein unfolding for long time scales.^36,37^ The wide range of amplitudes and time scales of conformational dynamics in such protease-sensitive protein segments suggests that the conformational dynamics are not themselves responsible for protease sensitivity; rather, it is the length of the disordered protein segment and spacing of its anchor points. Hubbard and Thornton modeled the deformability of potential trypsin cleavage sites in elastase, finding that the authentic cleavage site was able to adopt the largest number of trypsin-binding conformations and suffered the smallest energetic penalty of unfolding the native conformation.^38^

**Figure 1:**
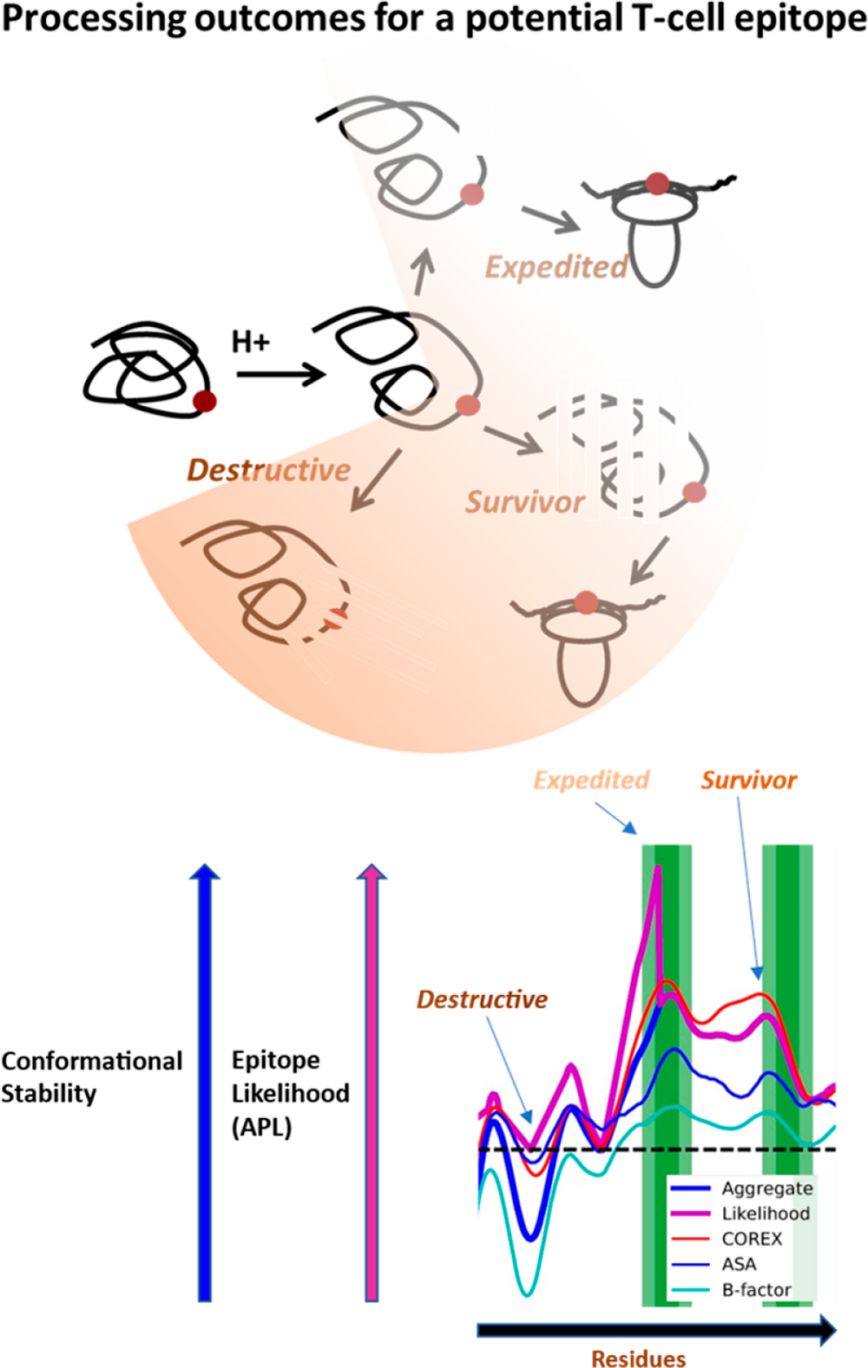
Mechanisms of CD4+ T-cell epitope dominance based on the relationship of the antigen conformation to proteolytic antigen processing. The filled red circle indicates the central residue of a potential epitope. The red shading indicates diminishing immunogenicity.

The potential for structure to influence peptide presentation was analyzed for individual proteins using an algorithm for antigen processing likelihood (APL). APL generates an aggregate profile of conformational stability in proteins using solvent-accessible surface area, crystallographic *b* factors, and COREX residue stabilities derived from three-dimensional structures, as well as sequence conservation, and then assigns epitope probability to conformationally stable segments with amplification for stable segments adjacent to unstable, putatively protease-sensitive segments (Figure 1, “Expedited” processing). Other stable segments may be immunogenic simply because they resist proteolysis long enough to be loaded into MHCII (“Survivor” processing). Unstable segments tend to be destroyed by proteolysis (“Destructive” processing). For a benchmark of 12 diverse non-self-antigens that were systematically mapped in human subjects, the accuracy of CD4+ epitope prediction achieved a value of 0.71 in the area under the curve (AUC) of sensitivity versus false positivity and reached a positive-predictive value of 43% for epitopes at the 95th percentile of the APL score.^39^ Accuracy was further improved when both APL and MHCII binding affinity were taken into account.

### Naïve T-Cell Frequency

The magnitude of the T-cell response has been shown to correlate with the size of the corresponding naïve T-cell population and the affinity of TCR for the peptide–MHC complex. The T-cell frequency is anticorrelated with self-antigen abundance, and this is presumably mediated by the abundance of peptide presentation.^40^ A growing number of studies connect the breadth and specificity of T-cell immunity with the composition of normal flora, potentially creating an enormous source of variation and environmental influence.^41^ Moreover, examples of T-cell priming by similar sequences in heterologous sources have long been considered mechanisms for autoimmunity, allergy, and protection.^42−46^

## Unstable and Metastable Viral Surface Proteins

Hypervariability is well established as a mechanism of immune evasion, wherein the direct benefit of protein sequence variation is predicated on repeated or chronic exposure to the pathogen (or related pathogen) because the exposure to a variant of the original pathogen will fail to be recognized as repeat exposure. In this context, hypervariable sequences typically coincide with disordered protein segments that are not constrained by the close packing of amino acid side chains.^47,48^ Hypervariability has frequently been identified in antigen sequences that present solvent-accessible surfaces on viruses and other pathogens, such as adenovirus, influenza virus, coronavirus, hepatitis C virus, the protozoan parasite *Plasmodium falciparum*, and the eubacterium group B *Streptococcus*.^47−51^

Antigen conformational disorder may also contribute to immune evasion in the context of a single exposure through conformational masking.^52^ In HIV Env, conformational masking was identified as conformational instability that creates a thermodynamic barrier to antibody binding. Kwong et al. used calorimetry to characterize the entropy cost of ligand binding at the CD4 (viral receptor)-binding site. The entropy cost affects both CD4 and antibody binding, but the virus retains CD4 binding by using multivalent avidity for multiple receptors on the cell surface. This could be demonstrated by the binding of the Env protein to a polymeric version of CD4 that mimics multiple CD4 molecules on the cell surface. Only very rare, highly affinity-matured antibodies can overcome the conformational flexibility in the CD4-binding site and bind with sufficient affinity to neutralize the virus. Other viruses may take advantage of the same phenomenon. In the context of conformational masking, hypervariability is an ancillary benefit arising from the lack of a well-folded structure.

Conformationally unstable protein domains attract weak T-cell responses due to destructive antigen processing and poor presentation of corresponding epitopes.^53−55^ Poor presentation could be evident as a lack of epitope immunogenicity, or the epitopes could be primed but then not recalled by secondary APCs, such as B cells or macrophage.

For the RNA virus envelope glycoproteins, proteolytic activation steps and conformational changes also support immune invasion. In type I viral fusion proteins, such as HIV Env, influenza hemagglutinin, and coronavirus spike proteins, the receptor-binding domain(s) and fusion machinery reside in N- and C-terminal fragments, respectively, that result from a proteolytic cleavage generally occurring prior to host cell association.^56−58^ In the prefusion conformational state, the fusion machinery is sequestered from antibodies in the protein interior and then emerges after target cell association. Following cell association, the N-terminal fragment dissociates and the C-terminal fragment catalyzes membrane fusion. Because the fusion machinery is more strongly conserved than the receptor-binding domain(s), this strategy reduces the level of exposure of the virus to cross-reactive antibodies induced by prior infections with related viruses. This strategy requires that the prefusion conformational state be a metastable state, effectively poised to undergo a conformational shift upon binding to the receptor and exposure to a low pH.

In addition to the conformational changes associated with membrane fusion, conformational changes that activate receptor binding have been characterized in multiple type I and type II fusion proteins, including HIV Env, influenza HA, coronavirus envelope glycoproteins, and hepatitis C virus.^56,59−61^ These changes are thought to support the evasion of neutralizing antibodies directed against the conserved receptor-binding site. For example, this mechanism explains the plethora of monoclonal antibodies that block binding of CD4 to monomeric HIV gp120 and neutralize tier 1 strains of HIV but are unable to neutralize tier 2 strains. Surfaces of the CD4-binding site on monomeric gp120 are obscured in the native trimeric Env molecule. Further conformational changes expose a second binding site for the co-receptor, whose binding precedes the activation of membrane fusion by gp41.^62^ HIV Env exists in a slow conformational equilibrium that is weighted to the “closed” state that hides the co-receptor-binding site. Presumably, the mechanism effectively excludes antibodies because their affinity is too weak to trap the “open” state when it becomes available. The possibility that such conformational changes have an impact on antigen processing has not been investigated.

### Conformational Masking in HIV Env

**Figure 2:**
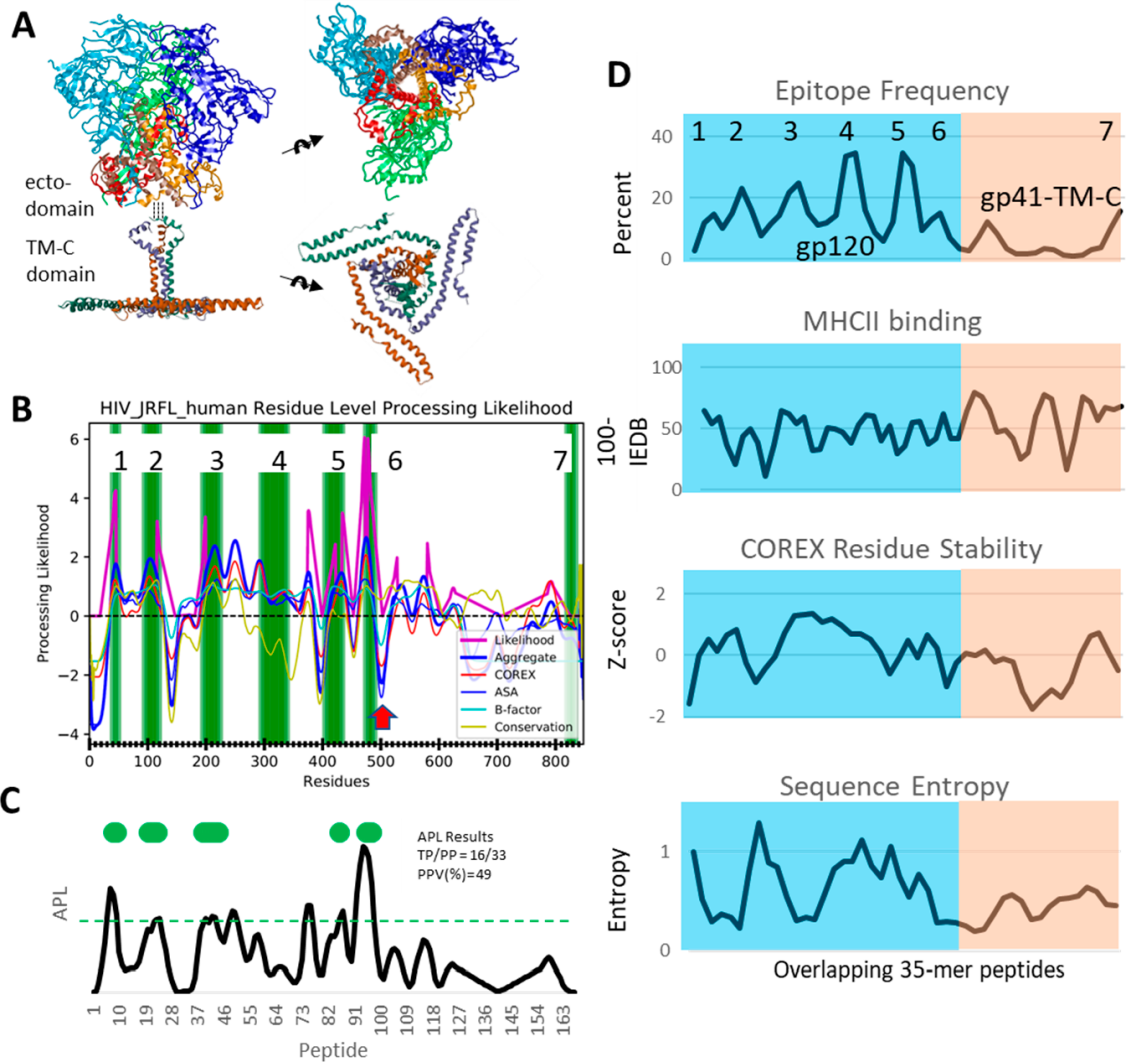
Conformational properties of HIV Env limit CD4+ T-cell immunity in the fusogenic gp41 and C-terminal tail. (A) Ribbon diagrams of the trimeric ectodomain (gp120 in blue, green, and cyan and gp41 in red, orange, and brown), transmembrane domain, and C-terminal tail (TM-C). (B) Stability and APL profiles of Env by residue. Red arrow indicates the gp120-gp41 cleavage site. (C) APL accuracy in predicting the 80th percentile of CD4+ epitopes reported in the IEDB. Abbreviations: TP/PP, number of true positive peptides divided by the number of positive predicted peptides; PPV, positive predicted value; D, comparison of immunologic profiles in Env fragments. Epitope frequencies are average IEDB residue frequencies for 35-mer peptides in 17-residue steps. MHCII binding is from the IEDB seven-allele method calculated for 15-mers and then averaged for five-peptide segments in three-peptide steps. Epitope clusters occur less frequently in gp41-TM-C, compared to gp120, in spite of increased MHCII binding affinity. Potential epitopes in gp41-TM-C may be destroyed by proteolysis, resulting in immune escape for the conserved sequences present. The composite structure of the HIV-1 envelope glycoprotein, strain JR-FL (Q75760), was constructed using the cryo-EM structure for the uncleaved ENV (PDB entry 7N6U) and a homology model based on the NMR structure (PDB entry 7LOI) of the strain HXB2 gp41 membrane-proximal external region, transmembrane domain, and cytoplasmic tail (TM-C).

As befits one of the deadliest infectious diseases, the HIV envelope glycoprotein (Env) has arguably been one of the most well-studied disease antigens, and it remains the most promising vaccine candidate for AIDS. Nevertheless, the extraordinary hypervariability arising from the virus’ low-fidelity reverse transcriptase and an array of other immune-evasive structural features have so far prevented the development of an effective vaccine. The Los Alamos National Laboratory (LANL) HIV Immunology Database has collected >200 CD4+ T-cell epitopes reported in studies with humans, mice, and other animals. In most cases, the restricting MHCII allele is not known. For some of the dominant epitopes, six or more alleles have been shown to present an epitope from the same protein segment. Epitope dominance is loosely defined across human studies because heterogeneous MHCII allele restriction yields epitope clusters of partially overlapping epitopes. Epitope frequencies are reported by LANL for each residue, and thus, we can sample the profile using a regularly spaced hypothetical peptide set (15-mers at five-residue step, 167 peptides) with the epitope frequency of the peptide defined as the average for all residues in the peptide. For the sake of discussion, dominant epitopes are defined as having a frequency in the 80th percentile across all peptides, which corresponds to an epitope frequency in the LANL database of 17%, i.e., triggering a response in 17% of sensitized subjects. At the 80th percentile, 34 dominant peptides are distributed in seven clusters (Figure 2). Using APL, we retrospectively predicted epitopes on the basis of the cryo-electron microscopy (cryo-EM) structure of the uncleaved Env ectodomain [Protein Data Bank (PDB) entry 7N6U] and an NMR-based model for the membrane-proximal and cytoplasmic domains (PDB entry 7LOI). Compared with the IEDB epitope profile, the positive predictive value (PPV) is a remarkable 48%. In other words, nearly half of the predicted 80th percentile peptides were observed as positive. This is a high success rate, especially considering that MHCII binding was not taken into account.

None of the seven CD4+ epitope clusters occur in gp41 or in the membrane span, and only one cluster occurs at the C-terminus of the cytoplasmic tail. Is this a real difference in the epitope frequency between regions of Env? Given that the clusters span an average of five peptides (35 residues), the clusters are oversampled; thus, the statistical comparison of the epitope frequency for gp120 versus gp41 and C-terminal domains (gp41-TM-C) is not appropriate with a peptide set of this density. A set of 35-mer peptides with 18-residue steps would sample the range of Env sequences in a series of peptides that match the frequency of epitope clusters. Using this peptide set, the gp41-TM-C has a significantly lower frequency of epitopes than gp120 (Table 1). The low frequency of dominant epitopes in the 300-residue gp41-TM-C is not due to the lack of MHCII binding capacity. In fact, the predicted binding of seven MHCII alleles thought to represent typical population binding capacity^9^ is significantly higher in gp41-TM-C than in gp120. In contrast, COREX residue stability^36^ is significantly lower in gp41-TM-C. Because conformational stability favors antigen presentation,^53^ gp41-TM-C could be less immunogenic because it is less stable and more sensitive to proteases.

**Table 1:** HIV Env Chain Properties (35-mer peptides)

	gp120	gp41-TM-C-term	*P* value
average epitope frequency (%)	16	4.8	0.00001
average MHCII binding (100-IEDB score)	46	56	0.01
average COREX residue stability (*Z* score)	0.31	–0.40	0.003
average sequence entropy	0.65	0.43	0.005

In addition to the neutralizing activity of an antibody binding to the Env trimer, other immune recognition events are important for protection and impose selective pressure on the Env protein sequence. Protective CD8+ T-cell epitopes are well-established in the context of certain class I HLA alleles, but most of these epitopes are located in the Gag gene product; their importance is limited to human populations bearing the relevant HLA alleles, and they are subject to rapid selection of resistant mutations.^63−65^ An antibody epitope in the V2 domain of Env has been investigated for its potential importance in protection by ADCC, but numerous animal studies and human trials so far have not provided a robust confirmation.^66,67^ In one of the few examples of association of disease with a CD4+ epitope, Ranasinghe et al. reported the positive correlation of viral load with the CD4+ T-cell response to a C2 epitope (corresponding to cluster 3 above).^68^ Mutation of nearby glycosylated residues has been shown to substantially modify presentation of gp120 and alter T-cell responses.^69^ Thus, although this epitope may be strongly dominant due to facile processing and presentation at priming, the outcome could be favorable to the virus if the recall response is blocked by a mutation at a nearby processing site. A more conventional negative correlation between viremia and CD4+ T-cell response was observed for three epitopes in the HIV Gag protein, for which the immunological mechanisms may be less entangled.^70^

### Influenza Hemagglutinin: Prototype of Conformational Misdirection

Influenza continues to take a harsh toll on humanity in spite of the long-standing and well-honed vaccination program. At any given time, several strains of the type A influenza virus are in global circulation. Typically, the strains express the H3 or H1 subtype of the hemagglutinin envelope glycoprotein (HA), which contains receptor-binding and membrane fusion activities. Although the virus does not rapidly mutate within individuals, reservoirs of the virus in pigs and birds periodically intersect with human populations to launch new viral strains that have acquired point mutations that disrupt neutralizing antibody epitopes and therefore support a new round of epidemic spread.^71^ Typically, a single genotype within a subtype dominates global infections in a given year. The gradual accumulation of a genetic change within a virus subtype is termed antigenic drift. More rarely, the virus acquires insertions or deletions that cause substantial structural changes and simultaneously disrupt multiple antibody epitopes. These genetic changes create different virus subtypes and are called antigenic shifts. Aside from H3 and H1, several additional subtypes, e.g., the “bird flu” bearing H5, are well represented among strains circulating in the animal reservoirs. We have previously noted that clusters of dominant epitopes coincide with hemagglutinin conformational domains and raised the possibility that deletions and insertions modify antigen processing, thereby substantially affecting the specificity of the CD4+ T-cell response.^72^ Within a subtype, T-cell responses may offer some protection when the dominant antibodies lose effectiveness due to mutations in the neutralizing epitopes, whereas the emergence in humans of an unusual subtype is a cause for alarm in the interest of public health.

Hemagglutinin is the prototype viral envelope glycoprotein that provides both receptor-binding and type I membrane fusion activities, which are performed by the HA1 and HA2 chains of the protein, respectively. The single hemagglutinin chain encoded by the viral genome must be activated for membrane fusion by a host protease, most commonly a trypsin-like activity in the secretory pathway of the cell that sheds the virus. Remarkable studies by Wiley and co-workers established that the trimeric hemagglutinin molecule undergoes an acid-induced conformational change in the stalk portion of the molecule that reveals the fusion peptide for interaction with the host cell membrane.^73^ Two α helices in each subunit of HA2 assemble with the intervening B loop to form a single long α helix. The exposed fusion peptide makes contact with the target membrane, and then, the trimer refolds into a six-helix bundle that helps bring the two membranes together. Crystal and/or cryo-EM structures are available for the uncleaved HA, cleaved HA1-HA2 at pH 5.0, and fusion-active HA2 molecules.^57^

**Figure 3:**
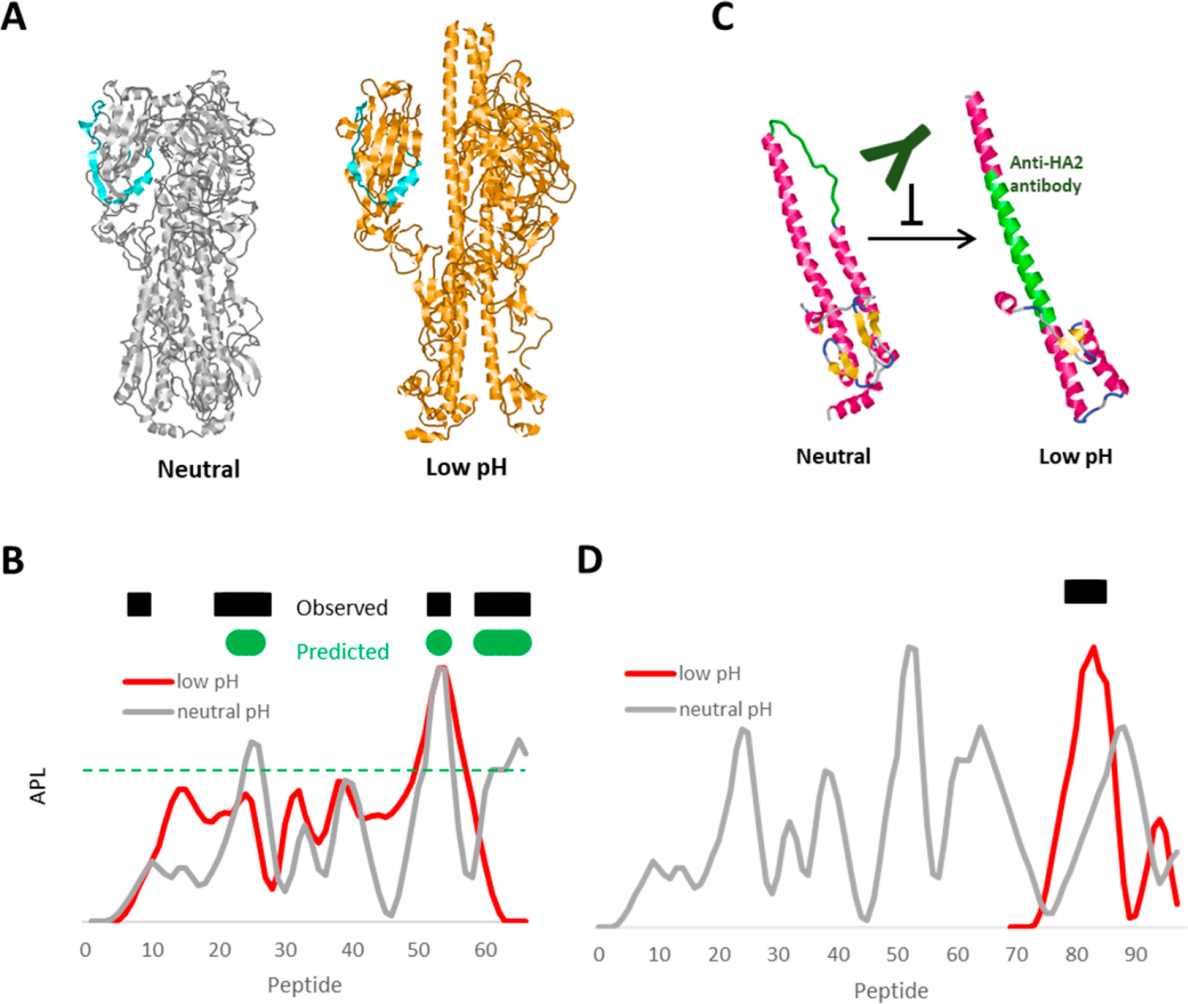
APL from neutral- and low-pH conformations of influenza HA1 and HA2. (A) Ribbon diagrams illustrating the low-pH conformational change in HA1 that reduces APL for peptides 22–27 (cyan), most likely by increasing their accessibility to proteolysis. (B) APL profiles for HA1 at neutral pH (gray) and low pH (red). Horizontal bars indicate dominant CD4+ epitopes (black) and peptides scoring at the 80th percentile in APL (green). (C) Conformations of a single HA2 subunit at neutral and low pH, illustrating the dominant T-cell epitope in the B loop (green ribbon segments). (D) APL profiles for HA at neutral pH (gray) and for HA2 at low pH (red). The horizontal black bar indicates a dominant epitope that is not presented by certain B cells that are specific for HA2.^74^ APL predicts dominant epitopes most accurately when based on the neutral-pH structure of HA1 and low-pH structure of HA2, which are the most stable conformations of each chain. For influenza hemagglutinin HA1, APL utilized the crystallographic structure (PDB entry 3LZG) for neutral pH and the cryo-EM structure (PDB entry 6Y5K) for low pH (except *b* factors from PDB entry 3QQI). For hemagglutinin HA2, APL utilized the crystallographic structures of PDB entry 3LZG for neutral pH and PDB entry 1HTM for low pH.

Distinct profiles of HA1 conformational stability at neutral and acidic pH yield distinct profiles of APL, and only the neutral profile matches the experimental epitope dominance (Figure 3B). Using the Immunome Browser tool at the Immune Epitope Database and Analysis Resource, we obtained a residue-by-residue profile of CD4+ epitope immunogenicity in influenza A hemagglutinin and then converted it into a regularly spaced series of 15-mer peptides as described above for HIV Env. Again, as discussed for HIV Env gp120 and gp41-Tm-C, we consider the possibility that the HA1 and HA2 chains exhibit distinct T-cell immunology and thus analyze them separately. At the 80th percentile of epitope frequency, 13 of 67 HA1 peptides score as dominant epitopes in two clusters of six (peptides 22–27 and 61–66) and two individual peptides (peptides 9 and 54). All four immunogenic segments are represented in mapping studies of the H3 and H1 hemagglutinin subtypes. APL captures three peptides in each of the clusters when using the neutral-pH structure (Figure 3B, gray line and green markers) but captures no peptides in either cluster when using the low-pH structure (Figure 3B, red line). Such a large swing in accuracy can be attributed to the destabilization of HA1 domains in the low-pH form of hemagglutinin. The cluster at peptides 22–27 spans an α helix that packs against HA2 in the neutral structure (Figure 3A). The cluster at peptides 61–66 spans a β strand that forms an antiparallel sheet with residues of peptide 9, and together, they have an extensive interface with HA2. At acidic pH, discharge of HA2 and disruption of the structure around these two peptide clusters are expected to make the epitopes sensitive to proteolysis. To account for their immunogenicity, we hypothesize that processing of most HA1 epitopes occurs in a dendritic cell compartment that remains above pH 5.0.

Characterization of T-cell clones from vaccinated subjects reveals a narrow CD4+ T-cell response and suggests that antigen processing can misdirect CD4+ T-cell help when the antigen undergoes a pH-dependent conformational change. Two regions of HA, one each in HA1 (peptides 52–54) and HA2 (peptides 80–82), have been identified as immunodominant for memory T-cell generation across multiple individuals with diverse MHCII allele compositions.^74^ Both epitope regions are represented by high-frequency TCR clones that distinguish them from subdominant epitopes. Among the four human subjects and five genetically defined MHCII proteins in this study, none of the MHCII molecules was shared between more than two subjects. Thus, dominance in these regions could not be adequately explained by the MHCII binding affinity, although the peptides are predicted to have nominal affinity for one or more of the available MHCII proteins. Studies using mass spectrometry of MHCII–peptide complexes from autologous B cells revealed that the dominance of both epitope regions is due to the high-level yield of the peptides from antigen processing and, in turn, presentation of the peptides to T cells. Interestingly, the dominant epitope in HA1 (peptide 54) is the one dominant peptide that was predicted well by APL using either the neutral- or low-pH structure.

In a remarkable example illustrating the influence of antigen processing on epitope selection, presentation by B-cell clones of the dominant epitope in HA2 was shown to be influenced by the specificity of the BCR.^74^ A B-cell clone specific for the HA1 domain presented essentially the same peptides as dendritic cells, as revealed by TCR sequencing, suggesting that processing in both B cells and DCs accessed the same conformation of HA2 (Figure 3C). In contrast, a B-cell clone specific for HA2 did not present the dominant epitope. Because some HA2-specific antibodies have been shown to block the acid-induced conformational change, it is likely that processing in the HA2-specific B cell could not access the conformation processed by DCs. In reviewing the neutral-pH structure of HA2, we see that the dominant epitope in peptides 80–82 is contained within the B loop segment, which could be sensitive to proteolysis, whereas in the acid-induced conformation of HA2, the B loop forms part of a long helix that is likely to be resistant to proteolytic destruction and preserves the epitope for presentation by MHCII. Details of how the epitope is extracted from this molecule remain to be discovered. Features of the antigen-processing mechanism may allow the gradual unfolding and sampling of potential epitopes in the MHCII groove, with segments of adequate affinity persisting through multiple rounds of DM-catalyzed exchange.

### CD4+ T-Cell Immunity to Flavivirus E Glycoproteins in Disease and Asymptomatic Infection

**Figure 4:**
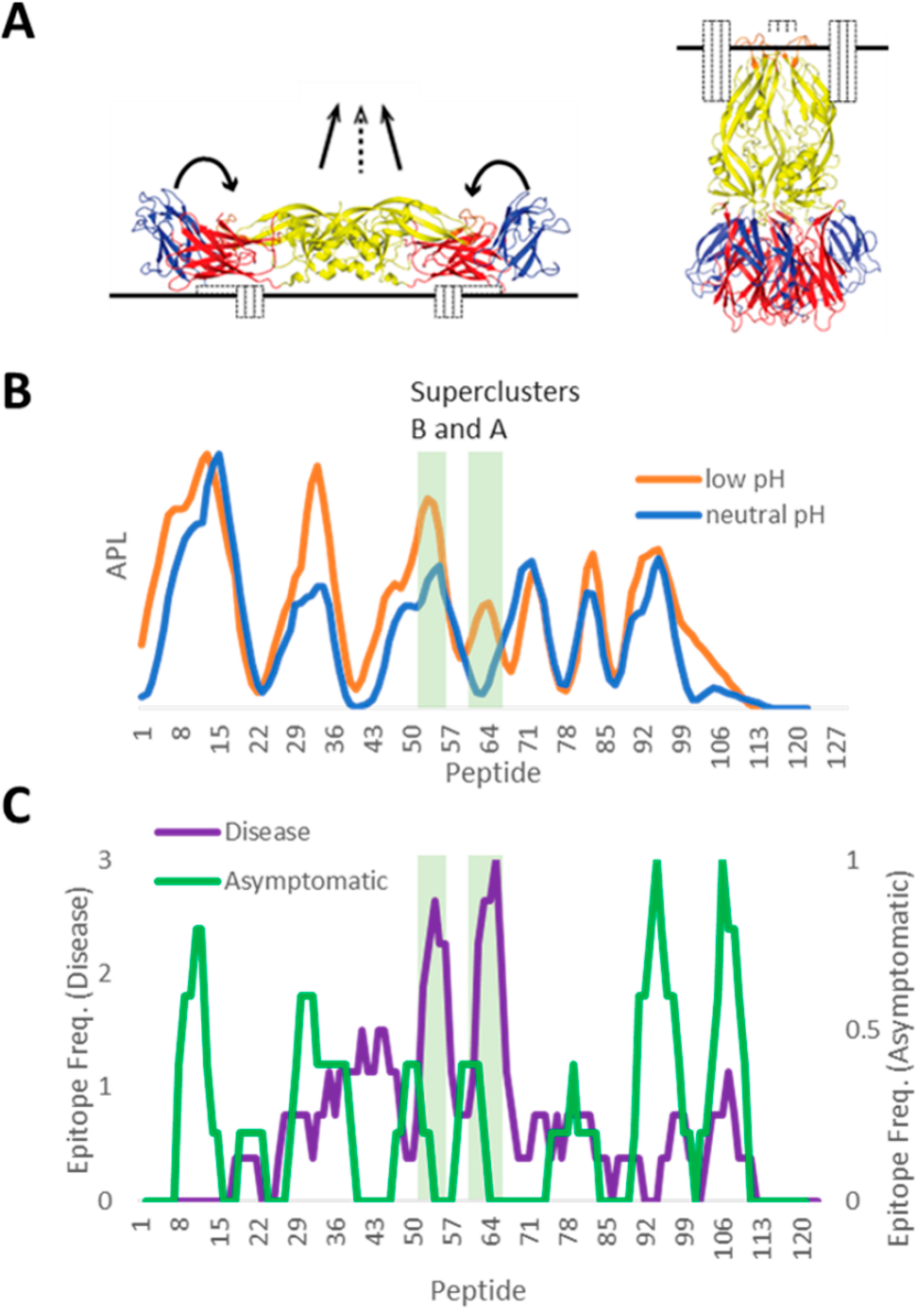
West Nile virus E protein structure and CD4+ epitope dominance. (A) WNV E membrane fusion conformational change. (B) APL for prefusion (blue) and postfusion (orange) conformations of WNV E protein, based on structures 7KV9 and 4GT0, respectively. (C) CD4+ epitope frequency reported by Koblischke et al.^78^ for 11 symptomatic subjects (violet) and 18 asymptomatic subjects (green). The profile of CD4+ epitope frequency in symptomatic disease is marked by increased immunogenicity of superclusters B and A, which are more prominent in APL generated with the postfusion conformation.

The flaviviruses represent a large family of enveloped viruses, including vector-borne Dengue fever virus (DFV), west Nile virus (WNV), Japanese encephalitis virus (JEV), tick-borne encephalitis virus (TBEV), Zika virus (ZV), and directly transmitted yellow fever virus and hepatitis C virus. Successful vaccines have been deployed for YF, JEV, and TBEV. The YF live attenuated vaccine is one of the most effective vaccines ever implemented. Flaviviruses display envelope E glycoproteins that possess receptor-binding and type II membrane fusion activities. Whereas the type I fusion proteins of HIV Env and Flu HA initially form a trimer pointing out from the viral membrane, the type II fusion proteins of flaviviruses initially form a dimer oriented with the long axis flat against the membrane and then, at acid pH, convert into a trimer pointed out from the viral membrane (Figure 4A). Rather than exposing a fusion peptide at the terminus of a proteolytic fragment, the type II fusion proteins expose a fusion loop that mediates contact with the host cell membrane.

CD4+ T-cell epitope dominance for E glycoproteins of TBEV, WNV, and ZV has been described as being strongly influenced by the protein conformation through its influence on antigen processing.^75−78^ A pattern of epitope clusters is shared across E proteins from vector-borne flaviviruses, and this pattern is predicted by APL according to the distribution of stable and unstable protein domains. The optimum accuracy of APL required use of the postfusion conformation of the E protein, and this dependence was observed for E glycoproteins from three viruses, DFV, JEV, and TBEV.^76^ Aberle and co-workers recently reported the CD4+ epitope dominance in 29 subjects infected with WNV,^78^ and again the postfusion structure was implicated. Of twelve 13-mer peptides that were identified as epitopes in at least two subjects, five were identified by APL using the postfusion structure, compared to only three when using the prefusion structure (data not shown). The most prominent increase in APL, associated with the pre-to-postfusion conformational transition, occurs for epitopes centered at residues 205 and 250 in WNV (Figure 4B, peptides 53 and 63), which coincide with superclusters B and A, respectively, in the aligned epitope maps of arbo-flaviviruses.^76^

Aberle and co-workers went a step further by tracking epitope usage in cohorts distinguished by disease severity.^78^ To account for epitope clustering and evaluate the sparse epitope data, the epitope maps were smoothed using a five-peptide moving window. Analyzed in this way, the difference between the asymptomatic and disease profiles are quite stark. Asymptomatic subjects exhibit the typical E protein profile, whereas the diseased subjects exhibit a profile with prominent peaks at residues 205 and 250 that match superclusters B and A, respectively (Figure 4C). Because epitopes at these two sites were more prominent in the postfusion structure, we conclude that severe disease coincides with T-cell priming of epitopes that are processed in more acidic lysosomes. Explanations include the potential enhancement of innate immunity secondary to increased pathology and the possibility that preexisting or excess inflammation favors processing and priming of the postfusion epitopes, which then cause or allow increased pathology.

### SARS-CoV-2 Spike in Vaccination and Infection

Having caused the deaths of more than 1 million people in the United States alone, COVID-19 stands apart as the most serious modern health threat that could be mitigated by vaccination. Unfortunately, more than half of these deaths occurred after effective vaccines became available because vaccine acceptance was weak, immunity waned quickly, and viral variants rapidly emerged. Explanations for immunity’s rapid wane and inadequate breadth remain unclear. Neutralizing antibodies are directed to the major envelope glycoprotein protein, spike, which is a type I fusion protein. As described for the original SARS-CoV spike, the mature spike is proteolytically cleaved by furin into the N-terminal S1 fragment and C-terminal S2 fragment that house receptor-binding and membrane fusion activities, respectively.^79,80^ Prior to binding the ACE2 receptor, the S1 fragment undergoes a conformational change in which the receptor-binding domain (RBD) rotates upward to expose the ACE2-binding site. Remarkably, this RBD-up conformation is disfavored at low pH, possibly to provide conformational masking that supports immune evasion.^81^ In a reaction promoted by ACE2 receptor binding, the S2 fragment is further cleaved at the S2′ site by transmembrane serine protease 2 (TMPRSS2) adjacent to the fusion peptide, and then the S1 fragment dissociates as part of the conformational change that displays the fusion peptide.^82^ On the basis of homology with other type I membrane fusion proteins, the acidic environment of the endosome is thought to promote the conformational change in S2.

**Figure 5:**
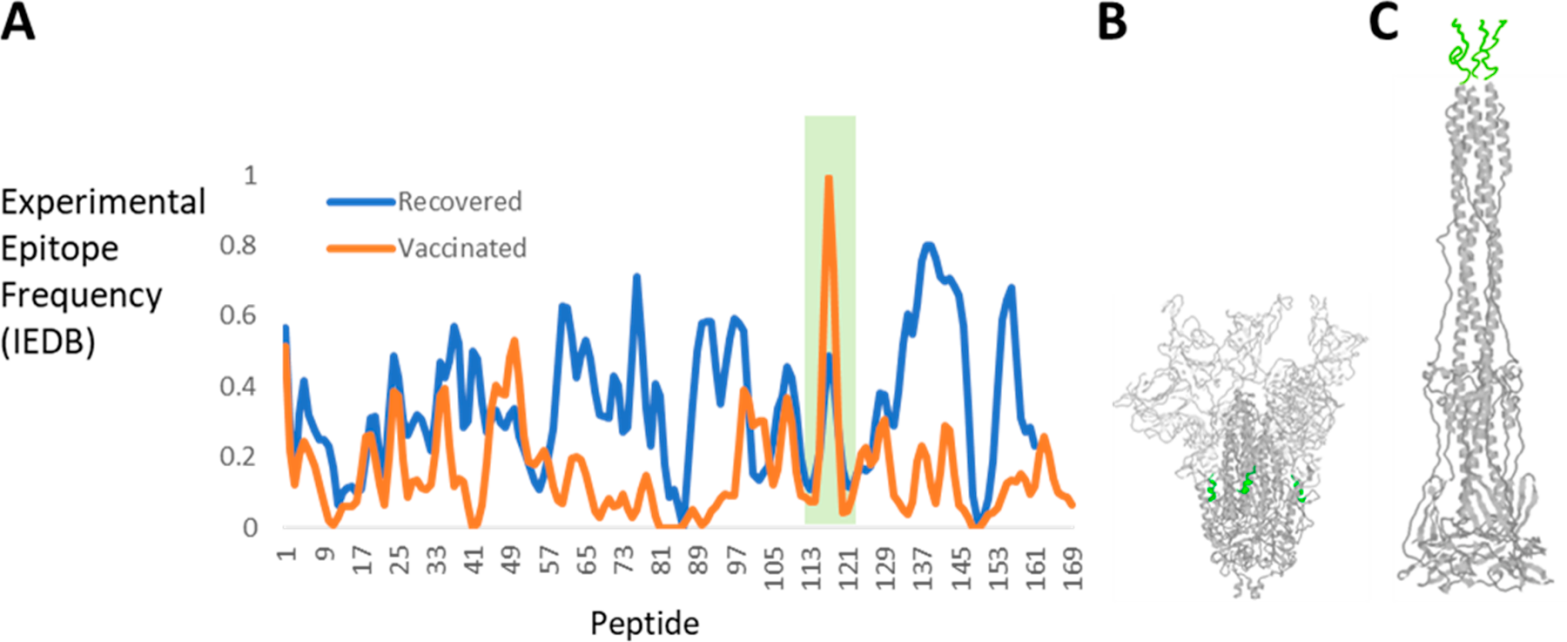
SARS-CoV-2 fusion peptide immunogenicity following infection and vaccination. (A) Plots of normalized epitope frequency in persons recovered from COVID infection or following mRNA vaccination. Residue-level frequencies reported in the IEDB were normalized by average frequency across all residues (infection, 32%; vaccination, 12%) and then averaged for residues in 17-mer peptides available from BEI Resources. (B) The 3-up conformation of SARS-CoV-2 spike (PDB entry 7DCC) with the S1 portion in white and the S2 portion in gray and fusion peptide (peptide 117) in green. (Antibodies that stabilize this conformation have been omitted for the sake of clarity.) (C) Postfusion conformation of SARS-CoV-2 spike S2 domain, modeled on the cryo-EM structure of the mouse hepatitis virus spike (PDB entry 6B3O). The epitope frequency for the fusion peptide is 6-fold above average following vaccination and 1.5-fold above average following infection. Reduced immunogenicity following infection may be due to destructive processing of the fusion peptide in the postfusion conformation.

CD4+ T-cell epitope dominance in SARS-CoV-2 spike depends on whether the exposure was by infection or vaccination. A recent examination of the IEDB found 22 studies on infected subjects reporting 313 epitopes and 14 studies on vaccinated subjects reporting 152 epitopes. To facilitate discussion of immunodominance, the residue-by-residue scores were averaged for 17-mer peptides spanning the spike ectodomain (using the same register as the peptide set from BEI Resources). CD4+ T-cell responses for infection are very broad, with 33 of the 161 peptides affording a response in at least 50% of tested subjects (Figure 5A). In contrast, the response to vaccine is narrower, with only 18 peptides affording a response in at least 10% of the tested subjects. The immunodominance patterns for infection and vaccination are substantially different. Among dominant epitopes from each route of exposure (those 32 peptides above the 80th percentile), only four peptides are shared. While the response to infection covers broad swaths of the spike sequence, the response to vaccination concentrates in several clusters of the S1 N-terminal domain (NTD) and in S2, especially at the C-terminal flank of the S2′ cleavage site (peptide 117). Prior exposure to the other human coronaviruses (HCoV) has been examined for an explanation of epitope dominance in spike. Homologous sequences from HCoV could have primed T cells that react with SARS-CoV-2, but dominant epitopes do not coincide with sequence similarity very well; therefore, the hypothesis was rejected.^83^ One well-conserved epitope in the fusion peptide seems to be the exception, and certain other SARS-CoV-2-reactive epitopes could have been primed by altogether different foreign antigens, as discussed below.

An understanding of CD4+ epitope dominance in spikes should first take into account the affinity of peptides for available MHCII proteins. The IEDB’s “seven-allele” method achieves a significant ROC AUC of 0.67 for vaccine-primed epitopes, indicating that the MHCII affinity influences epitope selection. However, the seven-allele method could not predict the dominant (IEDB 80th percentile) epitopes with significant accuracy, suggesting that MHCII affinity exerts a stronger influence at lower thresholds. Tarke-Sette found that the 49 most dominant epitopes from COVID-19 cases were characterized by higher HLA binding promiscuity, being able to bind approximately twice as many different HLAs as could a set of 49 non-epitopes.^83^ These results are consistent with MHCII binding having an influence but not controlling dominance.

Analysis of peptide presentation by mass spectrometry, also known as immunopeptidomics, offers a path to analyzing processing and presentation that bypasses the requirement for T-cell recognition. Eluted peptides are typically quantified by the number of overlapping peptides, i.e., the complexity of peptides containing a given sequence. Two studies have undertaken immunopeptidomics of SARS-CoV-2 spike peptides presented by monocyte-derived dendritic cells, one using the original virus spike with a single amino acid substitution at the furin cleavage site and the other using a two-proline-stabilized spike with the sequence “GSAS” at the furin site.^84,85^ Both discover similar peptide clusters, with some notable differences, and both studies yield peptide profiles that match the vaccine-induced CD4+ dominance profile to the extent of ∼67%.

Within S2, differences in CD4+ epitope dominance profiles from infection and vaccination can be rationalized by the transition to the postfusion conformation. Peptides 99 (S1/S2 C-terminal flank), 108, and 117 (fusion peptide) are at the 90th percentile of epitope frequency in vaccinees but are below the 80th percentile in recovered subjects, whereas peptides 137–145, 155, and 156 in the helical repeats are at the 50th percentile or below in vaccinees and 90th percentile in recovered subjects. This pattern is consistent with fragmentation and loading of the prefusion S2 in vaccinees and postfusion S2 in infected subjects. The stabilized prefusion conformation protects epitopes around the S1/S2 junction and fusion peptide (Figure 5B), and the postfusion structure protects the helical repeats (Figure 5C), resulting in presentation of the respective epitopes in vaccinees and recovered subjects, respectively.

Bulk sequencing of T-cell receptor genes allows for the measurement of T-cell clone frequencies after stimulation with intact antigen and therefore has the potential to reveal epitope hierarchies that reflect antigen processing. A number of dominant TCRs have been identified in recovered or vaccinated individuals. Short-term T-cell lines were stimulated by intact spike^46,86^ or peptide pools^87,88^ followed by bulk sequencing or generation of T-cell clones. For one study with vaccinated subjects, lymph-node fine-needle aspirates were sequenced directly ex vivo.^89^ In many cases, the corresponding epitopes have been associated with multiple class II HLA molecules. In a handful of cases, “public” TCR sequences specific for the same epitope have been found in multiple individuals. Four of the public TCRs correspond to epitopes in S2 (largely overlapping BEI peptides 108, 117, 124, and 158). All except peptide 158 lie near the S1/S2 and S2′ cleavage sites, which may facilitate processing and presentation of these epitopes in the “expedited” manner of Figure 1.

The epitope in peptide 117 coincides with the N-terminus of S2′ and contains the N-terminal portion of the fusion peptide. TCRs specific for peptide 117 were present in 97% of tested vaccinees (mostly with a mRNA-type vaccine), and T-cell responses by vaccinees to this peptide were by far the most frequently reported in the IEDB.^90^ T cells for peptide 117 arise somewhat less frequently from infection. The robust response in vaccinees appears to arise from cross-reaction with T cells that were primed by human coronaviruses (HCoV), which have a very similar sequence (nine residues identical in segment 816–830). The T cells reacting to SARS-CoV-2 peptide 117 exhibit surface molecules associated with T-cell effector function; they are amplified upon infection or vaccination, and they have been implicated in protection against severe disease through T-helper mechanisms.^91^ The HLA molecules having the highest predicted affinity for peptide 117 include combinations of some of the most common alleles, HLA-DPA1*01:03 and HLA-DPB1*02:01/DPB1*04:01/DPB1*04:02.

Remarkably, the adenovirus-based ChAdOx1 nCOV-19 (Asta-Zeneca) vaccine did not recall cross-reactive T cells.^92^ Numerous differences in vaccine composition and route of delivery could explain a difference in the recall of cross-reactive T cells. Nevertheless, a prominent difference is the fact that the mRNA-coded spike was conformationally stabilized by two proline substitutions in S2 but this adenovirus-coded spike was not. In the stabilized spike, S2 is effectively blocked from accessing the postfusion conformation that extends the fusion peptide (containing peptide 117) toward the host cell membrane. The low recall of peptide 117-specific T cells by this adenovirus-based vaccine resembles the low recall of peptide 117 T cells by infection. We hypothesize that the conformationally stabilized spike protects peptide 117 from destructive proteolytic processing that would destroy the fusion peptide after the postfusion conformational change. Some support for this hypothesis is available from the immunopeptidomics studies of MHCII-displayed spike peptides, which also differed in the use of stabilized or unstabilized spike proteins. In the study by Parker et al. using stabilized spike, the single most prominent peptide cluster overlapped the fusion peptide, whereas in Knierman et al. using unstabilized spike, peptide clusters overlapping the fusion peptide were represented at no more than 30% of the level of clusters in S1 and the helical repeats of S2.^84,85^

### Prospects

The study of antigen processing is poised to drive major developments in understanding immunopathology and vaccine design. Epitope-specific responses that have long been the focus of mapping efforts now find renewed scrutiny at the level of tissues, APCs, and innate-immune circumstances. The rapid accumulation of structural information from cryo-EM studies and computational analyses will illuminate structure–immunology relationships that shape disease and evolution of the pathogen and host.

## Methods

### Epitope Frequency Data from Public Databases

The T-helper epitope map for HIV-1 gp160 (last updated April 19, 2022) was downloaded as the list by amino acid residue (HIV-1 strain HXB2) from the Los Alamos National Laboratory HIV molecular immunology database (https://www.hiv.lanl.gov/content/immunology/density_plots/helper_by_aa.txt). Values assigned to the HXB2 sequence positions were transferred to the aligned JRFL (Q75760) sequence positions. The MHC class II-restricted T-cell epitope map for influenza A hemagglutinin (H1N1, P03452) was downloaded July 28, 2022, from the Immunome Browser at the IEDB Web site. The epitope map was automatically generated using epitope peptides collected by the IEDB from 106 studies of human immune responses. The MHCII class II-restricted T-cell epitope maps for SARS-CoV-2 spike were downloaded on June 6, 2022, from the Immunome Browser at the IEDB Web site. Maps were generated using epitope peptides collected from 22 studies of humans recovered from COVID and 13 studies of humans that were vaccinated against COVID using the mRNA vaccines.

### Calculation of Antigen Processing Likelihood (APL)

APL profiles were generated using the algorithm with parameters optimized for human epitopes as described previously.^39^ The input stability data were weighted as follows: sequence entropy, 0.17; *b* factor, 0.18; COREX residue stability, 0.51; solvent-accessible surface area, 0.15. Algorithm parameters were set as follows: magnification, 2.4; flank size, 11 residues; and loop size, 16 residues. To determine the Shannon sequence entropy, 35–100 homologous sequences with a range of sequence identity of 30–95% were collected using BlastP and then aligned using ClustalW. The Shannon sequence entropy was calculated using the tool in BioEdit.^93^
